# Human Segmentation and Tracking Survey on Masks for MADS Dataset

**DOI:** 10.3390/s21248397

**Published:** 2021-12-16

**Authors:** Van-Hung Le, Rafal Scherer

**Affiliations:** 1 Department of Information Technology, Tan Trao University, Tuyen Quang 22000, Vietnam; 2 Department of Intelligent Computer Systems, Czestochowa University of Technology, 42-218 Czestochowa, Poland; rafal.scherer@pcz.pl

**Keywords:** MADS dataset, human segmentation, human tracking, convolutional neural networks

## Abstract

Human segmentation and tracking often use the outcome of person detection in the video. Thus, the results of segmentation and tracking depend heavily on human detection results in the video. With the advent of Convolutional Neural Networks (CNNs), there are excellent results in this field. Segmentation and tracking of the person in the video have significant applications in monitoring and estimating human pose in 2D images and 3D space. In this paper, we performed a survey of many studies, methods, datasets, and results for human segmentation and tracking in video. We also touch upon detecting persons as it affects the results of human segmentation and human tracking. The survey is performed in great detail up to source code paths. The MADS (Martial Arts, Dancing and Sports) dataset comprises fast and complex activities. It has been published for the task of estimating human posture. However, before determining the human pose, the person needs to be detected as a segment in the video. Moreover, in the paper, we publish a mask dataset to evaluate the segmentation and tracking of people in the video. In our MASK MADS dataset, we have prepared 28 k mask images. We also evaluated the MADS dataset for segmenting and tracking people in the video with many recently published CNNs methods.

## 1. Introduction

Human segmentation and tracking in the video are two crucial problems in computer vision. Segmentation is the process of separating human data from other data in a complex scene of an image. This problem is widely applied in recognizing the activities of humans in the video. Human tracking extracts the person’s position during the video and is applied in many tasks such as monitoring and surveillance.

The MADS dataset is a benchmark dataset for evaluating human pose estimation. This dataset includes activities in traditional martial arts (tai-chi and karate), dancing (hip-hop and jazz), and sports (basketball, volleyball, football, rugby, tennis, badminton). The fast execution speed of actions poses many challenges for human segmentation and tracking methods. In [1], the authors report on the results of human tracking in the video; however, the results are evaluated based on baseline tracking methods. Human tracking and segmentation evaluation are based on the segmented person data based on the constraint of the context. In the MADS dataset, only two sets of human mask data are provided (tai-chi and karate).

The human data needs to be segmented and tracked in the video to reduce the estimated space and computations in human pose estimation in 2D images or 3D space. Specifically, to reduce the space to estimate 3D human pose on the 3D data/point cloud in future studies, we have prepared manually masked data of humans in the single view (depth video) of various actions (jazz, hip-hop, sports). Nowadays, with the strong development of CNNs, many studies have applied CNN methods in the task of estimating, detecting, and tracking humans. Human tracking in the video can be done based on two methods. The first method is based on human detection in each frame, the results of human being detected and marked with a bounding box. The CNNs (Faster R-CNN [2], SSD [3], YOLO [4,5,6], etc.) used to detect humans were surveyed in the study of Xu et al. [7] and Tsai et al. [8]. The second method to track humans is motion-based [9]. In this paper, we survey studies that use CNNs to segment and track humans in the video. We manually prepared the masked data for the MADS dataset (MASK MADS). We also fine-tuned a set of parameters on the depth videos of the MADS dataset. Moreover, we trained and evaluated human segmentation and tracking by various state-of-the-art CNNs.

**Figure 1:**
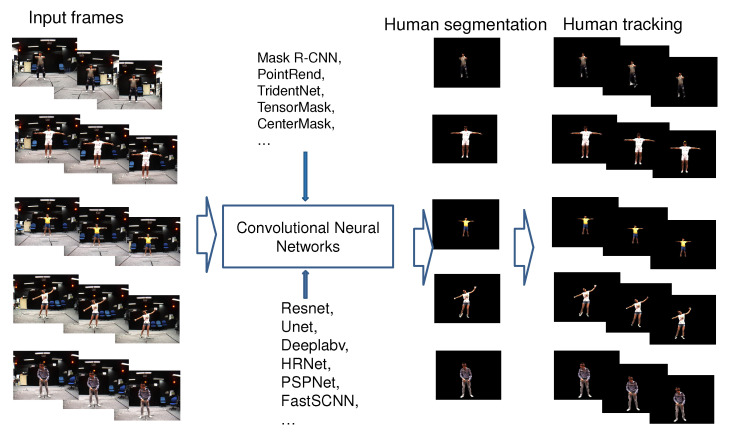
Illustrating of the process of segmenting and tracking humans in image sequences and video.

The main contribution of the paper is as follows:

- We manually prepared human masks for nearly 28 k images captured from a single-view. The marking process was performed using the interactive-segmentation tool (http://web.archive.org/web/20110827170646/, http://kspace.cdvp.dcu.ie/public/interactive-segmentation/index.html (accessed on 18 April 2021)).
- We summarised significant studies on human segmentation and human tracking in RGB images, in which we focus on survey studies that use CNNs for human segmentation and tracking in video. Our survey process is based on methods, datasets, evaluations, and metrics for human segmentation and human tracking. We ultimately analyze the challenges during the process. We also refer to the implementation or the source code path of each study. In particular, we investigate the challenges and results in human segmentation and human tracking in images and video.
- We fine-tuned a set of parameters of the recently published CNNs (Mask R-CNN, PointRend, TridentNet, TensorMask, CenterMask, etc.) to retrain the model for human segmentation and tracking on the video that was captured from single-views of the MADS dataset, as illustred in Figure 1. The data of the MADS dataset was divided into different ratios for training and evaluation.
- We evaluated the results of human segmentation in images based on the retrained CNN models (Mask R-CNN, PointRend, TridentNet, TensorMask, CenterMask) according to the data rates of the MADS dataset. We used the most significant CNNs in recent years for object segmentation.

The paper is organized as follows. Section 2 discusses related works by the methods, results of the human segmentation, and tracking. Section 3 presents the survey of human segmentation and tracking methods based on CNNs, and Section 4 our MASK MADS dataset. Section 5 shows and discusses the experimental results of human segmentation and tracking on state-of-the-art CNNs, and Section 6 concludes the paper.

## 2. Related Works

Human segmentation and tracking in video are highly applicable in activity recognition and surveillance. Therefore, these two issues have spurred research interest for many years. Especially in the past five years, with the advent of CNNs, their performance improved significantly in terms of processing time and accuracy. Since then, many studies have been investigated these two issues based on CNNs.

In the research of Xu et al. [10], the authors performed a survey of segments of human data in images obtained from still cameras using CNN. In their work, a person is detected and marked with a bounding box in their work. In this study, the authors also presented the method to segment the human data based on detecting humans in images. Among these, there are some awe-inspiring results of CNNs: Fast R-CNN [11], Faster R-CNN and YOLO, etc. The precision of SSD300, SSD512, YOLO on the Pascal VOC 2007 is reported 79.4%, 82.5%, 63.5% [12], respectively. The processing speed of Faster R-CNN and YOLO is 10 fps and 45 fps, respectively. In this study, the authors are also presented pixel-level segmentation of human data that uses CNNs for fine-tuning. The CNNs are introduced as Fully Convolutional Network [13], AlexNet [14], VGGNet [15].

Yao et al. [9] conducted an overall survey of methods and challenges in object segmentation and tracking in video. They divided the methods for segmenting and tracking objects into five groups as follows: unsupervised methods for object segmentation, semi-supervised methods for object segmentation, interactive methods for object segmentation, weakly supervised methods for object segmentation, and segmentation-based methods for object tracking. The authors also presented challenges in the process of object segmentation and tracking in video, namely: complex background of the scene, low resolution, occlusion, deformation, motion blur, and scale variation. The authors attempt to present approaches to answering questions such as: What is the application context of segmentation and audience tracking? What form is the object represented (point, superpixel, pixel)? What are the features that can be extracted from the image for object segmentation and tracking? How to build an object’s motion model? Which datasets are suitable for object segmentation and tracking? Although the authors have introduced datasets and metrics for the evaluating of subject tracking in the video, the results on these datasets have not been presented.

Ciaparrone et al. [16] performed a surveyed of deep learning-based multi-object tracking in the video captured from a single camera. The authors have presented the methods, measurements, and datasets of multi-object tracking. Therein, the used methods for multi-object tracking are presented in the following direction: object detection for tracking. According to the objects, the stages of the process are listed as follows: object detection with output marked with the bounding box, appearance feature extraction to predict the motion; computing a similarity between pairs of detections; assigning an ID for each object. In this study, the authors also presented metrics that evaluate the tracking of objects in the image: an evaluation based on trajectories, ground-truth trajectories are marked on the frames in the video; evaluation based on Jaccard similarity coefficient based on accuracy, precision, and recall of the detected, labeled bounding box of each object [17]. In [1], the authors were followed by the Bayesian tracker algorithm [18] and twin Gaussian processes algorithm [19] for multi-view tracking; Personalized Depth Tracker [20] and Gaussian Mixture Model tracker [21]. Although these methods are the start of human tracking, many CNNs have recently been introduced to solve this problem. We will introduce them in the next section.

The process of detecting, segmenting, and tracking people in the video is sequential in computer vision. To track people in videos, people need to be detected and segmented in each frame; to segment people in videos at the pixel level, people’s data must be detected and marked with a bounding box. As described above, each step of detecting, segmenting, and tracking people in videos is surveyed in our study. To provide an overview of the whole process, we conducted a survey covering all three stages: detecting, segmenting, and tracking people in the video.

In our future studies, we will use human mask data to segment human point cloud (3D point) data with the scene, supporting the estimation and evaluation of 3D human pose estimation. The point cloud data of a human is generated based on depth data and color data a human segmented from a human mask. The process of converting to point cloud data is done [22]. Each 3D point (*P*) is created from a pixel with coordinates (x,y) on the depth image and a corresponding pixel on the color image that has a color value C(r,g,b). *P* includes the following information: coordinates ($P_{x},P_{y},P_{z}$) in 3D space, the color value of that point ($P_{r},P_{g},P_{b}$), where the depth value (*D*) of point P(x,y) must be greater than 0. *P* is computed according to the Formula (1).

(1) $$\begin{array}{cc} P_{x} & =\frac{(x-c_{x})*D}{f_{x}}\\ P_{y} & =\frac{(y-c_{y})*D}{f_{y}}\\ P_{z} & =D\\ P_{r} & =C_{r}\\ P_{g} & =C_{g}\\ P_{b} & =C_{b} \end{array}$$

where ($f_{x}$, $f_{y}$—focal length), ($c_{x}$, $c_{y}$—center of the images) are intrinsics of the depth camera.

There are now many CNNs for estimating 3D human pose from human point cloud data such as HandpointNet [23], V2V [24], Point-to-Point [25]. Especially, there are many studies on 3D human pose estimation with amazing results on depth image data and point cloud data [26,27,28]. These studies have been examined in detail in our previous study on 3D human pose estimation [29]. In the future, we will study more deeply about using point cloud data in the MASK MADS dataset.

## 3. Human Segmentation and Tracking by CNNs—Survey

### 3.1. CNN-Based Human Segmentation

#### 3.1.1. Methods

**Figure 2:**
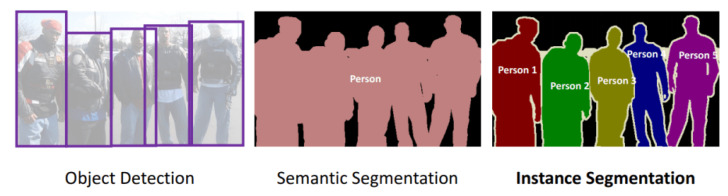
An explanation of object detection and semantic and instance segmentation [30].

**Figure 3:**
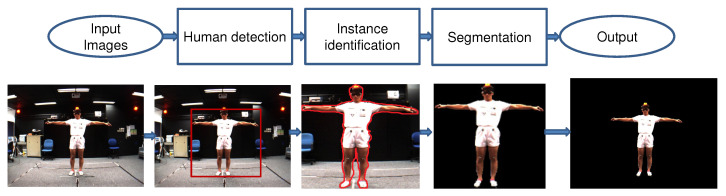
Illustration of the model of human segmentation in images.

Human segmentation is applicable in many practical and real-world scenarios, for example, surveillance person activities, virtual reality, action localization, 3D human modeling, etc. Human segmentation is the process of separating human data and scene data [10]. The methods for segmenting human data can be divided into three directions: top-down (semantic segmentation), bottom-up (instance segmentation), and combined. The top-down methods are based on training human-extracted features (shapes, appearance characteristics, and texture) to generate a classification model to classify the human pixels and scene pixels. This family of methods only segments persons into one class; despite there being many people and cars in the image, people are classified into one class, cars into another class. The bottom-up methods are based on generating the candidate regions that include a human and then identifying these regions following texture and bounding contours. Thus they segment the details of each person in the image. Figure 2 shows the differences between the approaches for human segmentation in images. The combined methods synergistically promote the advantages of both top-down and bottom-up methods to obtain the best effect. The human segmentation process is usually based on three steps described later on, as illustrated in Figure 3.

*a. Human detection*

Object detection, and specifically human detection in images or videos, is one of the most important problems in computer vision. During appearance-based methods, traditional machine learning often uses hand-crafted features and a classification algorithm (e.g., SVM, AdaBoost, random forest, etc.) to train the human detection model. In recent years, most of the studies and applications have used CNNs to detect persons and objects in general and demonstrated many impressive results.

Girshick et al. [31] proposed a Region-based Convolutional Neural Network (R-CNN) for object detection. This network can be applied as a bottom-up method for localizing and segmenting objects of region proposals and improved classification efficiency by using supervised pre-training for labeled training data. He et al. [32] proposed SPPnet (Spatial Pyramid Pooling network) to train the object detection model. Traditional CNNs include two main components: convolutional layers and fully connected layers. To overcome the fixed-size constraint of the network, SPPnet adds an SPP layer to the last convolutional layer. The fixed-length output features are generated from the SPP layer pools. SPP is robust with object deformations. The extracted features of variable scales are pooled by SPP. Karen et al. [15] based their assumptions on the characteristics of CNNs that the depth of the CNNs affects the accuracy. The greater the depth, the greater the identification detection accuracy. Therefore, the authors have proposed the VGG16 network with the input size of the convolutional layer of 224 × 224 RGB image. After that, the input image passed a stack of convolutional layers. The final output size of the convolutional layer is 3 × 3. Recently, Xiangyu et al. [33] improved the VGG model in Fast R-CNN for object classification and detection; Haque et al. [34] also applied the VGG model to ResNet to detect objects. Implementation details of VGG for object detection are shown under the links (https://www.robots.ox.ac.uk/~vgg/research/very_deep/, https://neurohive.io/en/popular-networks/vgg16/ (accessed on 20 May 2021)). To improve the results of R-CNN and SPPnet, Girshick et al. [11] proposed Fast R-CNN, which input is the entire image and a set of region proposals. Fast R-CNN performs two main computational steps: processes several convolutional and max-pooling layers on the whole image to generate a feature map. Each proposal interest region of the pooling layer then extracts a fixed-length feature vector from the generated feature map, and the input of a sequence of fully connected layers is the extracted feature vector. Implementation details of Fast R-CNN for object detection are shown under link (https://github.com/rbgirshick/fast-rcnn (accessed on 25 May 2021)). The SPPnet [32] and Fast R-CNN [11] models work on region proposals that could be the object, which reduces the computation burden of these CNNs. However, the accuracy of these networks has not been greatly improved. Ren et al. [2] proposed an RPN (Region Proposal Network) that shares the full-image convolutional features with the detection network, which makes a nearly cost-free region proposal. The architecture of Faster R-CNN consists of two parts: a deep, fully convolutional network (RPN) and a Fast R-CNN detector that uses the proposed regions. Implementation details of Faster R-CNN for object detection are available under link (https://towardsdatascience.com/faster-r-cnn-object-detection-implemented-by-keras-for-custom-data-from-googles-open-images-125f62b9141a (accessed on 10 July 2021)). Especially recently, Goon et al. [35] used Faster R-CNN for detecting pedestrians from drone images. The CNNs presented (R-CNN, SPPnet, VGG, Fast R-CNN, Faster R-CNN) are mainly concerned with the high accuracy, but the computation time for object detection is high. Therefore, Redmon et al. [4] proposed the YOLO network with a computation speed of about 67 fps of YOLO version 2 on the VOC 2007 dataset. The bounding boxes are predicted directly using the fully connected layers on top of the convolutional feature extractor. Currently, the YOLO network has four versions (YOLO version 1 to 4). Implementation details of YOLO versions for object detection are available under the link (https://pjreddie.com/darknet/yolov1/, https://pjreddie.com/darknet/yolov2/, https://pjreddie.com/darknet/yolo/ and https://github.com/AlexeyAB/darknet (accessed on June 2021)), respectively. Lui et al. [3] proposed the Single Shot Detector (SSD) network for object detection. It uses the following mechanism: the base network is used for high-quality image classification, fixed-size bounding boxes and scores are generated from a feed-forward convolutional network, and the final detections are generated by a non-maximum suppression step (https://github.com/weiliu89/caffe/tree/ssd (accessed on 12 June 2021)). Jonathan et al. [36] have performed a comparative study for objects detection, which focuses on comparing object detection results-based on typical CNNs: Faster R-CNN [2], R-FCN [37], and SSD [3]. The CNNs used the feature extractors as VGG or ResNet, calling them “meta-architectures”. The authors evaluated many configurations of each CNN and analyzed the effect of configurations, image size on the detection results.

*b. Human segmentation*

The next step in the model shown in Figure 3 is human segmentation, which is the process of labeling each pixel as either human or non-human data. It uses the result of object detection in the form of bounding boxes or identifies the human region, and then classifies the pixels in the bounding box or the area as human or non-human giving the most accurate results [10]. Previously there were studies by Meghna et al. [38,39] that suggested human pose-based segmentation. Lately, much research was proposed based on deep learning, for example, He et al. [40]. In [40], the authors proposed Mask R-CNN using Faster R-CNN for object detection and predicting an object mask on each Region of Interest (RoI) conducted in parallel, in which predicting a segmentation mask in a pixel-to-pixel basis. Implementation details of Mask R-CNN are available under link (https://github.com/matterport/Mask_RCNN (accessed on 14 June 2021)), and as Detectron2 (https://github.com/facebookresearch/detectron2 (accessed on 14 June 2021)) [41]. In the Detectron2 toolkit from Facebook AI Research [41], the authors also developed source code to train and test the segmentation of the image on some CNNs models: DeepLabv3 [42,43], details are shown in link (https://github.com/facebookresearch/detectron2/tree/master/projects/DeepLab (accessed on 12 June 2021)). DeepLabv3 is the human semantic segmentation group, this CNN is an improvement of the DeepLab2 [44] method. This method has been applied in parallel to the Atrous Spatial Pyramid Pooling (ASPP) method for multi-scale context. In [42], the authors improved the DeepLabv3 network with a combination of the spatial pyramid pooling module and the encoder-decoder structure. Therein, the rich semantic features are obtained from the encoder module; the detected objects by the bounding boxes are recovered by the decoder module. This network architecture of DeepLabv3+ made a trade-off between precision and processing time based on the extracted encoder features by atrous convolution. DensePose [45,46], details are shown in link (https://github.com/facebookresearch/detectron2/tree/master/projects/DensePose (accessed on 12 June 2021)). Riza et al. [46] proposed DensePose-RCNN for estimating human pose. DensePose-RCNN is a combination of the DenseReg and the Mask-RCNN to improve the accuracy, with the cascaded extensions. Cheng et al. [47,48] proposed the Panoptic-DeepLab (details are shown in the link (https://github.com/facebookresearch/detectron2/tree/master/projects/Panoptic-DeepLab (accessed on 14 June 2021)), which predicts the semantic segmentation and instance segmentation based on the dual-context and dual-decoder modules. The ASPP is employed in the decoder module. Kirillov et al. [49] proposed the PointRend method, details are shown in link (https://github.com/facebookresearch/detectron2/tree/master/projects/PointRend (accessed on 14 June 2021)). The PointRend network is applied in both semantic segmentation and instance segmentation. It was applied to each region in the coarse-to-fine method (from large to small size). Chen et al. [50] proposed a TensorMask network applied to the instance segmentation. Before the sliding window method used for object detection, the results are displayed on bounding boxes. After that, they use Mask R-CNN for object segmentation on the data inside in bounding box. Details are shown in link (https://github.com/facebookresearch/detectron2/tree/master/projects/TensorMask (accessed on 20 June 2021)); Li et al. [51] proposed a parallel multi-branch architecture called the TridentNet with ResNet-101 backbone retrained, details are shown in link (https://github.com/facebookresearch/detectron2/tree/master/projects/TridentNet (accessed on 15 June 2021)). This CNN used the image with multi-scales as the input. After that the image pyramid methods are used for feature extraction and object detection for each scale. Especially recently, the Centermask network is proposed and published by Lee et al. [52], this CNN implemented the human instance segmentation, details are shown in link (https://github.com/youngwanLEE/CenterMask (accessed on 16 June 2021)). This net-work used the anchor-free object detector (FCOS) [53,54] to predict the per-pixel object detection. After that, the SAG-Mask branch was added to predict a segmentation mask on each detected box. The feature extractions and feature map used the pyramid method of VoVNetV2 [55] backbone network.

George et al. [56] proposed the PersonLab model to estimate human pose and segment human instance from the images. This model used CNNs to predict all key points of each person in the image, after that the author predicted instance-agnostic semantic person segmentation maps by a greedy decoding process to group them into instances. This means that the determination of an *i*th human pixel is based on the probable distance from that pixel to the nearest detected keypoint. Implementation details are shown in link (https://github.com/scnuhealthy/Tensorflow_PersonLab (accessed on 16 June 2021)). Zhang et al. [57] have proposed a model based on the human instance segmentation method where the object detection step is based on the results of human pose estimation. The human pose is estimated based on the combination of scale, translation, rotation, and left-right flip, and it is called Affine-Align. The Affine-Align operation uses human pose templates to align the people which does not use a bounding box as in Faster R-CNN or Mask R-CNN, in which the human pose templates are divided into clusters and center of clusters used to compute the error function with detected poses by the affine transformation. The human segmentation module is concatenated from the Skeleton features to the instance feature map after Affine-Align. Implementation details are available under link (https://github.com/liruilong940607/Pose2Seg (accessed on 18 June 2021)).

#### 3.1.2. Datasets, Metrics and Results

*a. Human detection*

Object detection in images and videos is the first operation applied in computer vision pipelines such as object segmentation, object identification, or object localization. Object detection methods have been evaluated on many benchmark datasets. In this section, we present some typical benchmark datasets.

Everingham et al. [58] introduced the Pascal VOC (PV) 2007 with 20 object classes with 9963 images which are collected from both indoor and outdoor environments. The interesting objects are divided into the following groups: person; animal (bird, cat, cow, dog, horse, sheep); vehicle (airplane, bicycle, boat, bus, car, motorbike, train); indoor (bottle, chair, dining table, potted plant, sofa, tv/monitor). This data set is divided into 50% for training/validation and 50% for testing. The dataset can be downloaded from link (http://host.robots.ox.ac.uk/pascal/VOC/voc2007/ (accessed on 19 June 2021)). In [59], the authors updated the Pascal VOC dataset (PV 2010) with 10,103 images containing 23,374 annotated objects and 4203 segmentations. In this dataset, the authors changed the way of computing the average precision and used all data points rather than TREC style sampling. In 2012, the authors updated the PV 2012 dataset [60] with the training/validation data of 11,530 images containing 27,450 ROI annotated objects and 6929 segmentations. These versions of the PV dataset are presented, compared in link (http://host.robots.ox.ac.uk/pascal/VOC/voc2012/ (accessed on 18 June 2021)).

In [61], Lin et al. published the benchmark MS COCO (Microsoft Common Objects in COntext) dataset. It includes 328,000 images with 80 common object categories and 2,500,000 labeled instances. The objects in the images are person, car, elephant and the background is grass, wall, or sky. After six years, this dataset is now available with more than 200,000 images and 80 object categories, and over 500,000 object instances segmented.

The ImageNet Large Scale Visual Recognition Challenge 2014 detection [62] task involves 200 categories. There are 450 k/20 k/40 k images in the training/validation/testing sets. The authors focus on the task of the provided data-only track (the 1000-category CLS training data is not allowed to use).

Most of the research on object detection presented above use the mean Average Precision (mAP) measurement for evaluation. It is calculated according to Equation (2).

(2) $$mAP=\frac{\sum_{Q}^{q=1}AverageP\left(q\right)}{Q}$$

where *Q* is the number of frames and AverageP(q) is the Average Precision (AP) of object detection for each frame. Precision is calculated as in [17]. Some results of human detection are shown in Table 1 and Table 2.

*b. Human segmentation*

Human data segmentation is the process of classifying whether each pixel belongs to a human object or not. To evaluate the human segmentation, the studies of Kaiming et al. [40] and Zhang et al. [57] were evaluated on the COCO [61] dataset and introduced the above. The metric used to evaluate is AP (Average Precision), precision is calculated as shown in [17].

The results of the human segmentation on the PV 2007 benchmark are shown in Table 3. The processing time of Mask R-CNN, Pose2Seg for human segmentation is 5 fps, 20 fps, respectively. We also listed the results of object segmentation on the PV 2017 benchmark dataset with the validation/testing sets from [61], and they are presented in Table 4.

#### 3.1.3. Discussions

Table 1 shows the human detection accuracy based on some benchmark datasets such as PV 2007, PV 2010, PV 2012, COCO, or IC datasets. Table 2 shows the processing time of human detection on the PV 2007 dataset. The results of human detection can show us that accuracy increases the following time and that the processing time of human detection has reached real-time. We only surveyed on the PV 2007 dataset to agree on the dataset. Each different version of the PV dataset has a different number of images and complexity. So the processing time will be different. When compared to the same dataset, we can see the processing speed of CNNs (e.g, R-CNN, Fast R-CNN, YOLO, etc) for detecting humans. Our survey is based on an evaluation of CNNs for human detection using the TensorFlow framework on the PV 2007 dataset. A part of which we refer to in the survey of Sanchez et al. [64].

As presented above, the human detection results significantly affect the process of human segmentation. As shown in first table and second table of [56], the keypoint detection results of human pose on the COCO dataset are between 61% and 75% on the $AP_{M}$, $AP_{L}$ measurements, in the human segmentation step is between 47% and 62%. Therefore, the error from detecting humans is from 25% to 39%, and the human segment error is about 14%. These results also show that the segmentation of the human data on the COCO dataset still poses many challenges.

### 3.2. CNN-Based Human Tracking

#### 3.2.1. Methods

Human tracking is the process of estimating the movement trajectory of people in the scene based on the video captured by the scene. A tracker is a set of object labels and classifications between that object and other objects and the background [65]. Human tracking is the process of reusing the results of human detection or human segmentation on each frame of the video. From there, the person’s trajectory is drawn on the video. Watada et al. [65] performed a human tracking survey, which uses the results of human detection (which is the process of detecting points of interest in the human body). This detector is called a “*point detector*” and the second method is to use the results of the human segment in the image. In this paper, we are interested in studies using CNNs to track people in the video. Dina et al. [66] experimented with a method for tracking multiple persons in images using Faster R-CNN to detect people. The results of people detection in the image are shown as bounding boxes. The author used VGG16 with thirteen convolutional layers of various sizes (64, 128, 256, 512), two fully connected layers, and a softmax layer to build the Faster R-CNN. Javier [67] has generalized the object tracking problem in video, in which the author presented Siamese Network Algorithm (SNA) for object tracking. SNA assumes that the object to track always be unknown, and there is no need to learn it. SNA uses the CNNs to parallel object detection in the images, and then it computes the differences of the pairs of images. Implementation details are shown in link (https://github.com/JaviLaplaza/Pytorch-Siamese (accessed on 20 June 2021)). Gulraiz et al. [68] proposed a model of human detection and tracking that used Faster R-CNN to detect the human with five implementation steps. Then, they use more Deep Appearance Features (DAF) to track humans. Especially, the method provided the motion information and appearance information. The authors also presented the challenges of object tracking systems. The first one concerns real-time human tracking. There has been much research trying to achieve the goal of real-time tracking of people. The second is the identity switch in all frames or specific time duration. The third is the fragmentation problem when the person is not detected at some frames, which causes the person’s moving trajectory to be interrupted. Particularly, we proposed solutions to improve the detection results in some frames by using CNNs for human detection or just detecting parts of the person, such as the head or shoulder. To address identity switches, the authors suggested using the appearance, localization features, and the size of a human in frames or perhaps using facial recognition. The proposed system is better than both SORT and Deep SORT [69,70] in real-time scenarios for pedestrian tracking. Ejaz et al. [71] proposed a method for improving human detection and tracking accuracy in noisy and occluded environments. To improve the accuracy of human detection and classification, a softmax layer is used in the CNN model. Special tactics of enhancing learning complex data (data augmentation) are applied.

#### 3.2.2. Datasets, Metrics and Results

To evaluate the results of human tracking, Laura et al. [72] proposed a huge MOTChellange dataset, which combined of 22 different subsets. Some results of human detection in images of MOTChellange dataset before human tracking based on the CNNs are shown in Table 5.

However, Gulraiz et al. [68] were only interested in 10-minute videos of individuals with 61,440 rectangles of human detection. It is composed of 14 different sequences with proper annotations by expert annotators. This dataset collection camera is set up in multiple states (dynamic or static). The camera position can be positioned horizontally with people or lower. The lighting conditions are also quite varied: lighting, shadows, and blurring of the pedestrians are inconsistent. The processing times of human detection for various ResNets are shown in Table 6.

In [34], the authors evaluated the INRIA human dataset and Pedestrian parsing on surveillance scenes (PPSS) dataset. The authors used the INRIA dataset that includes 2416 images for training and 1126 images for testing. The persons in the INRIA dataset were captured from many different positions: pose and occluded background, crowd scenes. The PPSS dataset included a total of 3673 images captured from 171 videos of different scenes and 2064 images in this dataset that the people are occluded. Haque et al. [34] used 100 videos for training and 71 videos for testing. The results of human tracking on the INRIA dataset and PPSS dataset are shown in Table 7.

## 4. Human Mask of MADS Dataset

**Figure 4:**
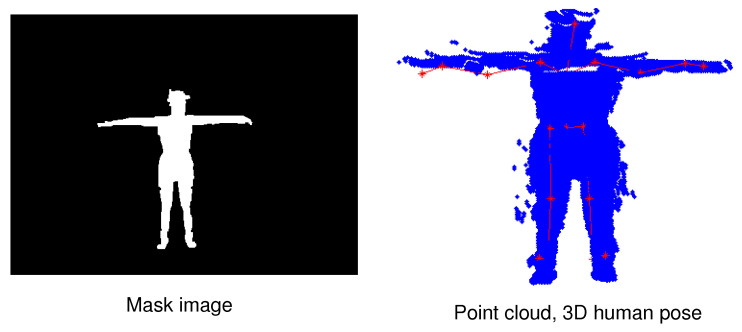
Illustrating of 3D human annotation data correction results according to point cloud data based on the human mask from the image. The red human skeleton is a result of 3D human pose estimation in the point cloud data.

**Figure 5:**
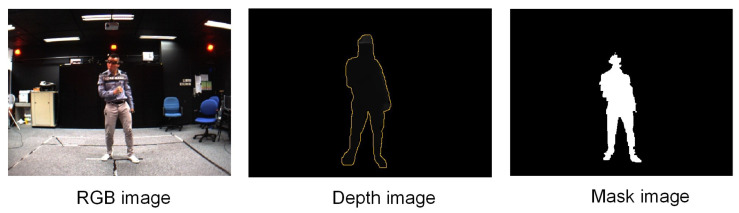
Illustration of image data of an unmarked human and masked image. Human depth data is delimited by a yellow border. The depth value of the human pixels is greater than 0 (which is the distance from the camera to the surface of the body) and is a gray color, the other pixels are the background and is black color. The depth image is the result of mapping from the human mask to the depth image obtained from the environment.

The MADS dataset includes martial arts (tai-chi and karate), dancing (hip-hop and jazz), and sports (basketball, volleyball, football, rugby, tennis, and badminton) actions. The activities in this dataset are fast and dynamic, and many body parts are active, especially arms and legs. The MADS dataset consists of two sets of data: The RGB image data collected from multi-view settings; RGB image data and depth image data collected from a single viewpoint (captured from the depth sensor). Figure 4 shows the mask image of the human (left) when segmented to the pixel level, and the point cloud data of a human (right) generated from the person data segmented on the depth image with the camera’s intrinsic parameter based on Equation (1). We also illustrate a result of 3D human pose estimation based on point cloud data (right). Therefore in this paper, we are only interested in the dataset collected from the depth sensor (the data is collected from a single viewpoint). We will use human point cloud data in further studies. An example of the RGB and depth data from the dataset is illustrated in Figure 5. To evaluate the results of human segmentation and tracking humans in videos, we implemented pixel marking of the human area in the RGB images that were captured from a single viewpoint (depth sensor).

To mask people in the image, we have manually prepared the mask data of the human using the Interactive Segmentation tool (http://web.archive.org/web/20110827170646/, http://kspace.cdvp.dcu.ie/public/interactive-segmentation/index.html (accessed on 18 April 2021)). We have prepared about 28,000 frames and make available at the link (https://drive.google.com/file/d/1Ssob496MJMUy3vAiXkC_ChKbp4gx7OGL/view?usp=sharing (accessed on 18 July 2021)).

## 5. Human Segmentation and Tracking of MADS Dataset

### 5.1. Methods

In this paper, we evaluate in detail the human instance segmentation on the MASK MADS dataset with the Mask R-CNN benchmark [40] method and some of its improvements in the Detectron2 toolkit [41].

**Figure 6:**
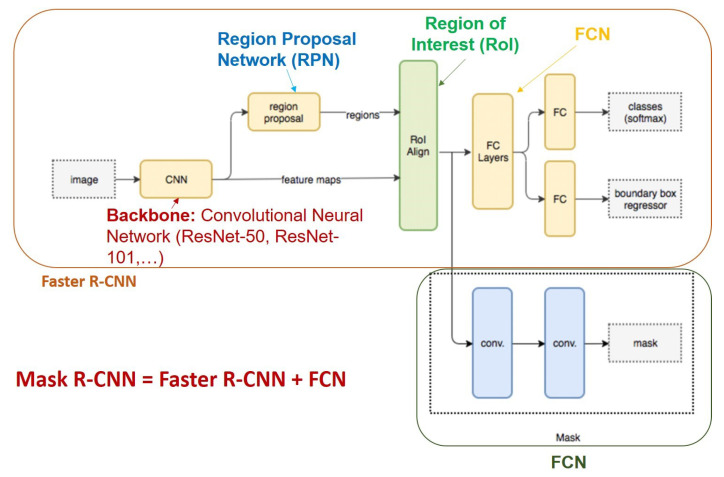
Mask R-CNN architecture human instance segmentation in images.

- Mask R-CNN [40] is an improvement of Faster R-CNN [2] for image segmentation at the pixel level. The operation of Mask R-CNN for human instance segmentation does the following several steps.
  Backbone Model: Using ConvNet like Resnet to extract human features from the input image.
  Region Proposal Network (RPN): The model uses the extracted feature applied to the RPN network to predict whether the object is in that area or not. After this step, bounding boxes at the possible areas of objects from the prediction model will be obtained.
  Region of Interest (RoI): The bounding boxes from the human detection areas will have different sizes, so through this step, all those bounding boxes will be merged to a certain size at 1 person. These regions are then passed through a fully connected layer to predict the layer labels and bounding boxes. The gradual elimination of bounding boxes through the calculation of the IOU. If the IOU is greater than or equal to 0.5 then be taken into account else be discarded.
  Segmentation Mask: Mask R-CNN adds the third branch to predict the person’s mask parallel to the current branches. Mask detection is a Fully-Connected Network (FCN) applied to each RoI. The architecture of the Mask-RCNN is illustrated in Figure 6.
  In this paper, we use Mask-RCNN’s code developed in [41]. The backbone model used is ResNet-50 and the pre-trained weights is
  “COCO-InstanceSegmentation/mask_rcnn_R_50_FPN_1x.yaml”.
  It is trained with ResNet-50-FPN on COCO *trainval35k* takes 32 h in our synchronized 8-GPU implementation (0.72 s per 16-image mini-batch) [40]. The code that we used for training, validation, testing is shared under the link (https://github.com/duonglong289/detectron2 (accessed on 10 June 2021)).
- PointRend [49]: PointRend is an enhancement of the Mask R-CNN for human instance and human semantic segmentation. This network only differs from Mask R-CNN in the prediction step on bounding-boxes (FCN), Mask R-CNN [40] performs the coarse prediction on a low-resolution (28×28) grid for instance segmentation, the grid is not irrespective of object size. However, it is not suitable for large objects, it generates undesirable “blobby” output that over smooths the fine-level details of large objects. PointRend predicts on the high-resolution output grid (224×224), to avoid computations over the entire high-resolution grid. PointRend suggests 3 strategies: choose a small number of real-value points to make predictions, extract features of selected points, a small neural network trained to predict a label from this point-wise feature representation of a point head. In this paper, the pre-trained weights that we use is
  “InstanceSegmentation/pointrend_rcnn_R_50_FPN_1x_coco.yaml”.
  That means the backbone we use is the ResNet-50. It is trained on the COCO [61] dataset with *train2017* (∼118 k images). The code that we used for training, validation, testing is shared in the link (https://github.com/duonglong289/detectron2/tree/master/projects/PointRend (accessed on 15 June 2021)).
- TridentNet [51]: TridentNet is proposed for human detection by bounding-box on images that are based on the start-of-the-art Faster R-CNN. TridentNet can improve the limitations of two groups of networks for object detection (one-stage methods: YOLO, SSD, and two-stage methods: Faster R-CNN, R-FCN). TridentNet generates scale-specific feature maps with a uniform representational power for training with multiple branches; trident blocks share the same parameters with different dilation rates. TridentNet training with ResNet-50 backbone on 8 GPUs, the pre-trained weights initialized in file “tridentnet_fast_R_50_C4_1x.yaml”. The code that we used for training, validation, testing is shared at the link (https://github.com/duonglong289/detectron2/tree/master/projects/TridentNet (accessed on 16 June 2021)).
- TensorMask [50]: TensorMask is an improvement of Mask R-CNN to use structured 4D tensors ((V, U) represent relative mask position; (H, W) represent the object position) to represent mask image content in a set of densely sliding windows. The dense mask predictor of TensorMask extends the original dense bounding box predictor of Mask R-CNN. TensorMask performs multiclass classification in parallel to mask prediction. The code we use has the pre-trained weights initialized in the file “tensormask_R_50_FPN_1x.yaml” and the ResNet-50 backbone on 8 GPUs are used. The code that we used for training, validation, testing is shared in the link (https://github.com/duonglong289/detectron2/tree/master/projects/TensorMask (accessed on 16 June 2021)).
- CenterMask [52]: CenterMask is an improvement of Mask R-CNN. During the implementation of Mask R-CNN, Centermask added a novel spatial attention-guided mask (SAG-Mask) branch to anchor-free one stage object detector (FCOS), the SAG-Mask branch predicts a segmentation mask on each box with the spatial attention the map that helps to focus on informative pixels and suppress noise. Although, in the present paper [52] used the backbone network VoVNetV2 based on VoVNet [55] to ease optimization and boosts the performance, that shows better performance and faster speed than ResNet [74] and DenseNet [75]. In this paper, we still use the pre-trained weights initialized in file “centermask_R_50_FPN_1x.yaml” The code that we used for training, validation, testing is shared in the link (https://github.com/duonglong289/centermask2 (accessed on 16 June 2021)).

Start-of-the-art Backbone: As discussed above, the backbone used to train the pre-train weights is ResNet-50.

**Figure 7:**
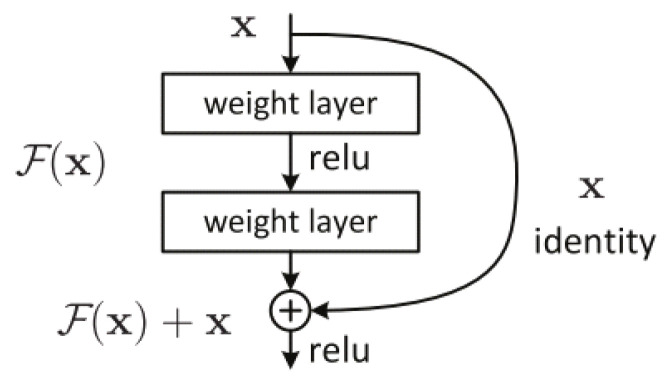
A Residual Block across two layers of ResNet.

ResNet is a very efficient deep learning network designed to work with hundreds or thousands of convolutional layers. When building a CNN with many convolutional layers, Vanishing Gradient will occur, leading to a suboptimal learning process. To solve the problem, ResNet proposes the idea of going from the output layer to the input layer and computing the gradient of the corresponding cost function for each parameter (weight) of the network. Gradient descent is then used to update those parameters. It is proposed to use a uniform “identity shortcut connection” connection to traverse one or more layers, illustrated in Figure 7. Such a block is called a Residual Block. The input of this layer is not only the output of the layer above but also the input of the layers that shorten to it.

**Figure 8:**
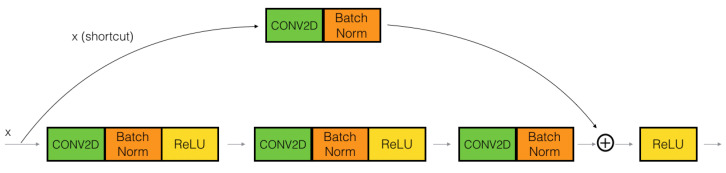
Illustrating convolutional block of ResNet.

In residual bock, the input *x* is added directly and the output of the network is F(x)+x, and this path is called an identity shortcut connection. The output of Residual Block is called H(x)=F(x)+x. So, when F(x)=0, then H(x)=x is said to be a homogeneous mapping when the input of the network equals its output. To add F(x)+x, the shape of both must be the same. If the shapes are not the same then we multiply a matrix $W_{s}$ by the input *x*. $H\left(x\right)=F\left(x\right)+W_{s}*\left(x\right)$, where $W_{s}$ can be trained. When the input of the network and the output of the network are the same, ResNet uses an identity block, otherwise uses a convolutional block, as presented in Figure 8.

Using residual blocks, in the worst case, the deeper layers learn nothing, and performance is not affected, thanks to skipping connections. But in most cases, these classes will also learn something that can help improve performance.

**Figure 9:**
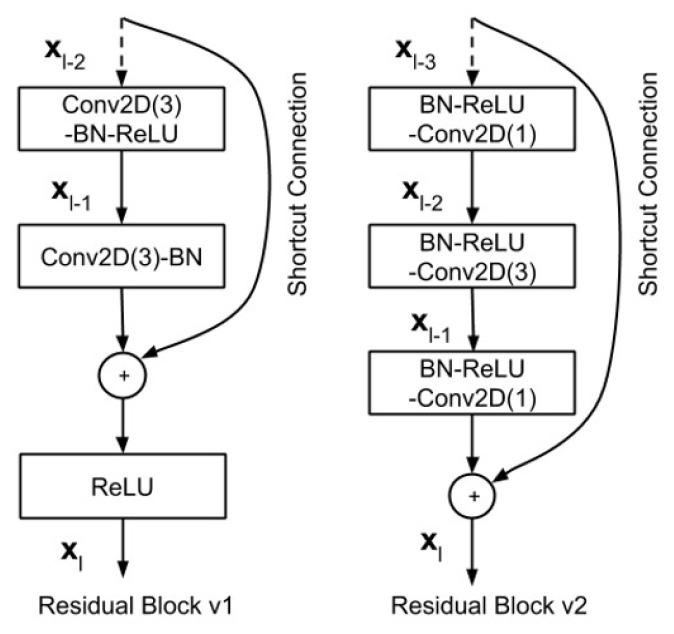
A comparison of residual blocks between ResNet v1 and ResNet v2 [77].

Recently, Resnet version 1 (v1) has been improved to ameliorate classification performance, and was called ResNet version 2 (v2) [76], where Resnet v2 has two changes in the residual block [77]: The use of a stack of (1 × 1) − (3 × 3) − (1 × 1) at the steps BN, ReLU, Conv2D, respectively; the Batch normalization, and ReLU activation that comes before 2D convolution. The difference between ResNet v1 and ResNet v2 is shown in Figure 9.

### 5.2. Experiments

To evaluate the results of human detection and human segmentation on the MADS dataset, we divide the MADS database into training and testing sets according to the following ratios: 50% for training and 50% for testing (rate_50_50), 60% for training, and 40% for testing (rate_60_40), 70% for training and 30% for testing (rate_70_30), 80% for training and 20% for testing (rate_80_20). The images are randomly assigned. The number of frames in the ratios is shown in Table 8.

In this paper, we used Colab Notebook with GPU Tesla P100, 16 GB for fine-tuning, training, testing the CNNs on the MASK MADS dataset. The processing steps, code fine-tuning, training, testing, and development process were performed in Python language (≥3.6 version) with the support of the OpenCV, Pytorch (≥3.6 version), CUDA/cuDNN libraries, gcc/& g++ (≥5.4 version), In addition, there are a number of other libraries such as Numpy, scipy, Pillow, cython, matplotlib, scikit-image, tensorflow ≥ 1.3.0, keras ≥ 2.0.8, opencv-python, h5py, imgaug, IPython. The parameters that we use are as follows: the batch size is 2, trained on 90 thousand iterations, the learning rate of 0.02; the weight decay is 0.0001, the momentum is 0.9, and other parameters are the same as when the default training of Mask R-CNN [40] and Detectron2 [41].

In this paper, we also use metrics of the standard COCO metrics including AP (Average Precision) of over IoU thresholds, $AP_{50}$, $AP_{75}$, and $AP_{S}$, $AP_{M}$, $AP_{L}$ (AP at different scales) [40].

### 5.3. Results

**Figure 10:**
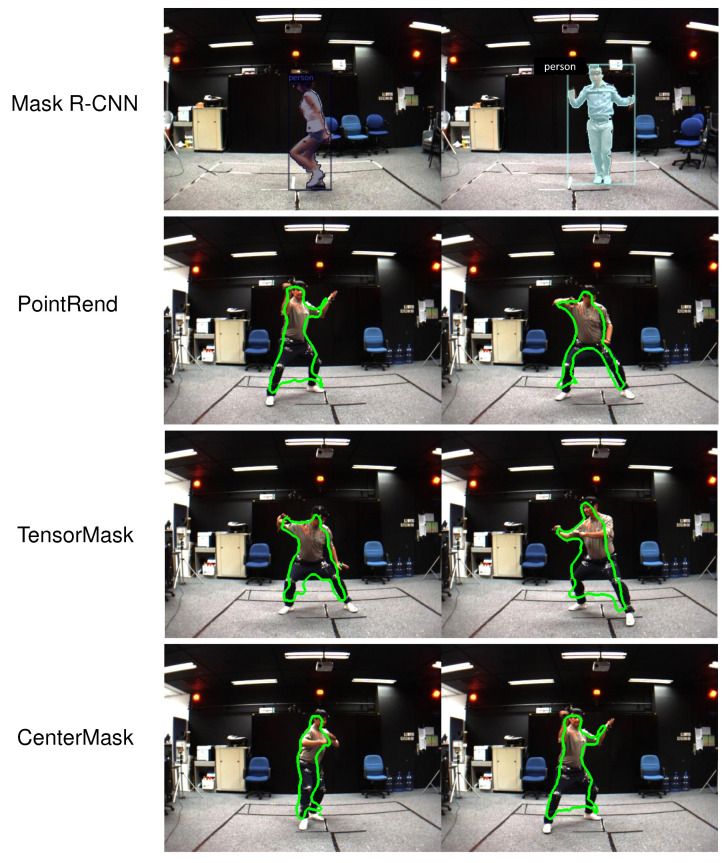
Examples of human segmentation results on the MADS dataset by CNNs.

**Figure 11:**
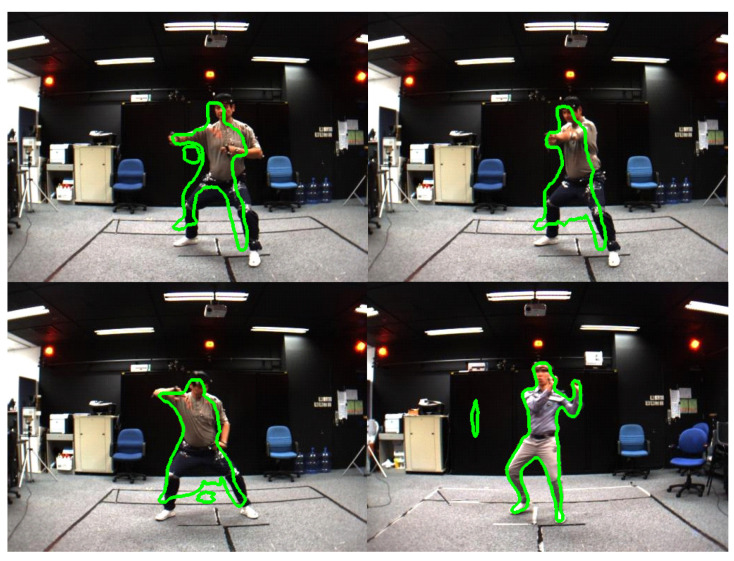
Examples of false human segmentation results on the MADS dataset.

We compare the results of human segments based on Mask R-CNN, PointRend, TridentNet, TensorMask, CenterMask on the MADS dataset, which have been divided by the ratios. The results based on box (*b*) are shown in Table 9. In Table 9 and Table 10, the human segmentation results on the box (*b*) and mask (*m*) of the CenterMask are the highest ($AP^{b}=69.47\%$, $AP^{m}=61.28\%$). Human segmentation results on the MADS dataset are also shown in Figure 10, and the wrong human segmentation results are also shown in Figure 11.

The results based on the mask (*m*) are shown in Table 10.

Figure 11 shows that there are a lot of wrongly segmented pixels (segmented background pixels of human data), and there are also some segmented areas of human data. The problem is the result of the wrong person detection step in the image. In this paper, we also have shared the complete revised source code of CNNs on links (https://github.com/duonglong289/detectron2.git), (https://github.com/duonglong289/centermask2.git), and the retrained model of CNNs on link (https://drive.google.com/drive/folders/16YHR8MxOn4l8fMdNCJZv56AcLKfP_K4-?usp=sharing (accessed on 16 June 2021)). Although there is only one person in the image of the MADS dataset (the data captured by the stereo sensor), it still poses many challenges. Due to the low quality of the images obtained from the stereo sensor, the images are blurred, the lighting is not perfect, and the activities of the people in the image are fast (martial arts, dancing, and sports), so the gestures of the legs and arms are blurred.

## 6. Conclusions

In this paper, we performed manual annotation of human masks in videos of data captured from a single view of the MADS dataset on 28 thousand images. This is called the Mask MADS dataset; it is shared for the community to use. We have conducted complete and detailed surveys on using CNNs to detect, segment, and track the people in the video. This survey goes from the mode of methods (CNNs), datasets, metrics, results, analysis, and some discussion. In particular, links to the source code of the CNNs are provided in this survey. Finally, we fine-tuned a set of parameters from the masked human data. We have represented the architecture of start-of-the-art methods and backbone model to fine-tune the human detection, segmentation model. We performed detailed evaluations with many recently published CNNs and published the results on the mask MADS dataset (Table 8, Table 9 and Table 10).

## Figures and Tables

**Table 1:** Human detection results (mAP) on the several benchmark datasets.

Measurement/ Dataset/Methods	*mAP* (Mean Average Precision) (%)						
	PV 2007	PV 2010	PV 2012	COCO (Year)	IC 2013	IC 2014	IC 2015
R-CNN [31]	58.7	58.1	-	-	31.4	-	-
SPPnet [32]	58.9	-	-	-	-	35.11	-
VGG vs. Fast R-CNN [15]	66.1	-	-	-	-	-	-
VGG vs. ResNet [34]	-	-	93.7	-	-	-	-
VGG 16 [15]	89.3	-	89	-	-	-	-
Fast R-CNN [11]	69.9	72.7	72.0	35.9 (2015)	-	-	42.9
Faster R-CNN [2]	78.8	-	75.9	42.7 (2015)	-	-	32.6
YOLO v1 [63]	63.4	-	63.5	-	-	-	-
YOLO v2 [4]	78.6	-	81.3	21.6 (2015)			
YOLO v3 [5]	-	-	-	60.6 (2015)	-	-	-
YOLO v4 [6]	-	-	-	65.7 (2017)	-	-	-
SSD300 [3]	74.3	-	79.4	23.2 (2015)	-	-	-
SSD500 [3]	76.8		83.3	26.8 (2015)	-	-	-

**Table 2:** The frame rate of human detection (fps—frames per second) on the PV 2007 benchmark dataset.

Measurement/ Dataset/Methods	Procesing Time (fps)
	PV 2007
R-CNN [31]	0.076
SPPnet [32]	0.142
VGG vs. Fast R-CNN [15]	7
VGG vs. ResNet [34]	5
Fast R-CNN [11]	0.5
Faster R-CNN [2]	7
YOLO v1 [63]	45
YOLO v2 [4]	40
YOLO v3 [5]	45
YOLO v4 [6]	54
SSD300 [3]	46
SSD500 [3]	19

**Table 3:** The frame rate of human detection (fps—frames per second) on the PV 2007 benchmark dataset. $AP_{M}$, $AP_{L}$ are the *Median*, *Large* AP categories, respectively.

Measurement/ Method/	Backbone	$AP_{M}$	$AP_{L}$
Mask R-CNN [40]	Resnet50-fpn	43.3	64.8
PersonLab [56]	Resnet101	47.6	59.2
PersonLab [56]	Resnet101(ms scale)	49.2	62.1
PersonLab [56]	Resnet152	48.3	59.5
PersonLab [56]	Resnet152(ms scale)	49.7	62.1
Pose2Seg [57]	Resnet50-fpn	49.8	67.0
Pose2Seg(GTKpt) [57]	Resnet50-fpn	53.9	67.9

**Table 4:** The object segmentation results (*m*—mask, *b*—box) on the COCO 2017 Val/Test dataset [61] (%—percent).

Measurement/ CNNs	Backbone Network	$AP^{m}$	$AP^{b}$	$AP_{S}^{b}$	$AP_{M}^{b}$	$AP_{L}^{b}$	IS	SS
CenterMask	VoVNetV2-99	38.3	43.5	25.8	47.8	57.3	*√*	-
TridentNet	ResNet-101	-	42.0	24.9	47.0	56.9	-	*√*
TensorMask	ResNet-101-FPN	-	37.1	17.4	39.1	51.6	*√*	-
PointRend (IS)	X101-FPN	40.9	-	-	-	-	*√*	*√*
Panoptic-DeepLab	Xception-71	39.0	-	-	-	-	*√*	*√*

**Table 5:** The results (accuaracy, precision (%)) of human segmentation on the MOTChallenge dataset. SNN is a human tracking evaluation based on the Siamese Neural Network. Ecdist is a human tracking evaluation based on the simple minimum Euclidean distance.

Measurement/Model	MOTA (MOT Accuracy) [%]	MOTP (MOT Precision) [%]
SORT [73]	59.8	79.6
Deep SORT [69]	61.4	79.1
Faster RCNN + DAF [68]	75.2	81.3
Faster RCNN [66] (Ecdist)	61.26	-
Faster RCNN [66] (SNN)	47.38	-

**Table 6:** The processing time (ms—millisecond) of Human detection for various ResNets. The calculation process is performed on a computer with the following configuration: GeForce GTX 1080 Ti GPU with Ubuntu OS installed in the system. The experiments are performed using the TensorFlow framework [68].

Measurement/Model	Processing Time [ms]
ResNet-34	52.09
ResNet-50	104.13
ResNet-101	158.35
ResNet-152	219.06
ResNet-30	48.93

**Table 7:** The results of Human segmentation on the INRIA dataset and PPSS dataset [34].

Dataset	Model	Accuracy [%]
INRIA dataset	VGG-16	96.4
	CNNs + DA [34]	98.8
PPSS dataset	VGG-16	94.3
	CNNs + DA [34]	98.3

**Table 8:** The number of frames in the ratios (%—percent) for training and testing of MASK MADS dataset.

Ratios (%)	The Number of Frames for Training	The Number of Frames for Testing
rate_50_50	14,414	14,414
rate_60_40	17,047	11,492
rate_70_30	19,141	8616
rate_80_20	21,473	5742

**Table 9:** The results (%—percent) of human segmentation (box-*m*) on the MADS dataset is evaluated on the CNNs.

CNN Model	Training/Testing Ratios (%)	$AP^{b}$ (%)	$AP_{50}^{b}$ (%)	$AP_{75}^{b}$ (%)	$AP_{S}^{b}$ (%)	$AP_{M}^{b}$ (%)	$AP_{L}^{b}$ (%)
Mask R-CNN [40]	rate_50_50	59.25	71.45	65.82	7.22	86.85	86.80
	rate_60_40	59.12	72.02	65.29	7.18	86.45	86.22
	rate_70_30	59.44	71.85	65.84	6.72	87.50	86.55
	rate_80_20	59.96	71.98	66.35	7.16	88.04	86.58
PointRend [49]	rate_50_50	63.25	78.98	72.19	8.03	67.89	84.71
	rate_60_40	64.58	79.81	72.87	9.64	68.84	85.78
	rate_70_30	67.90	81.40	74.30	13.82	72.11	88.69
	rate_80_20	66.67	80.91	73.53	11.29	71.74	87.52
TridentNet [51]	rate_50_50	61.17	70.84	65.89	6.42	91.39	88.77
	rate_60_40	55.50	71.80	66.25	0.75	50.81	79.66
	rate_70_30	61.28	70.87	65.85	5.71	91.53	88.86
	rate_80_20	61.50	70.96	66.67	6.18	92.01	88.23
TensorMask [50]	rate_50_50	67.11	80.95	74.15	11.78	69.58	88.49
	rate_60_40	67.41	81.71	74.01	12.36	73.79	87.67
	rate_70_30	64.37	79.81	72.38	7.93	67.09	85.74
	rate_80_20	64.78	80.20	73.13	8.95	69.69	85.63
CenterMask [52]	rate_50_50	65.94	79.78	73.09	8.39	71.59	87.75
	rate_60_40	64.75	79.89	72.08	9.21	68.03	86.24
	rate_70_30	65.40	79.36	71.57	6.77	68.72	87.95
	rate_80_20	**69.47**	**81.63**	**75.04**	**12.95**	**74.44**	**91.10**

**Table 10:** The results (%—percent) of human segmentation (mask-*m*) on the MADS dataset evaluated on the CNNs.

CNN Model	Training/Testing Ratios (%)	$AP^{m}$ (%)	$AP_{50}^{m}$ (%)	$AP_{75}^{m}$ (%)	$AP_{S}^{m}$ (%)	$AP_{M}^{m}$ (%)	$AP_{L}^{m}$ (%)
PointRend [49]	rate_50_50	58.73	78.86	69.17	9.35	59.70	80.63
	rate_60_40	59.39	79.26	68.48	10.81	55.64	81.07
	rate_70_30	62.66	81.23	70.70	14.11	60.39	83.55
	rate_80_20	61.93	80.58	70.48	11.48	63.29	83.17
TensorMask [50]	rate_50_50	54.10	79.38	65.71	8.48	52.30	74.35
	rate_60_40	57.08	80.01	67.97	10.23	55.36	77.26
	rate_70_30	47.71	77.97	55.07	5.88	38.55	69.53
	rate_80_20	50.87	78.39	60.42	6.70	44.74	72.12
CenterMask [52]	rate_50_50	53.43	79.18	65.85	8.47	56.48	71.71
	rate_60_40	52.24	78.69	64.28	8.80	53.35	70.93
	rate_70_30	52.67	78.96	64.60	7.19	54.36	71.27
	rate_80_20	**61.28**	**81.19**	**72.10**	**13.61**	**66.89**	**79.72**

## Data Availability

The data is available upon the request.
